# Persistence of *Penaeus stylirostris* densovirus delays mortality caused by white spot syndrome virus infection in black tiger shrimp (*Penaeus monodon*)

**DOI:** 10.1186/1746-6148-9-33

**Published:** 2013-02-15

**Authors:** Sudkhate Molthathong, Sarocha Jitrakorn, Yutthana Joyjinda, Chuenchit Boonchird, Boonsirm Witchayachamnarnkul, Pattira Pongtippatee, Timothy Flegel, Vanvimon Saksmerprome

**Affiliations:** 1 Department of Biotechnology, Faculty of Science, Mahidol University, 10400, Bangkok, Thailand; 2 Centex Shrimp, Faculty of Science, Mahidol University, 10400, Bangkok, Thailand; 3 National Center for Genetic Engineering and Biotechnology (BIOTEC), National Science and Technology Development Agency (NSTDA), Thailand Science Park, 12120, Pathum Thani, Thailand; 4 Shrimp Genetics Improvement Center, Surat Thani, Thailand; 5 Aquatic Animal Biotechnology Research Center, Surat Thani Campus, 84100, Surat Thani, Thailand

**Keywords:** PstDNV, IHHNV, Shrimp, WSSV, Real-time PCR, Multiplex PCR

## Abstract

**Background:**

Persistent infection of *Penaeus stylirostris* densovirus (PstDNV) (also called IHHNV) and its non-infectious inserts in the black tiger shrimp, *Penaeus monodon (P. monodon*) genome are commonly found without apparent disease. Here, we introduced the method of multiplex PCR in order to differentiate shrimp with viral inserts from ones with the infectious virus. The method allowed us to study the effect of pre-infection of IHHNV, in comparison to IHHNV inserts, on WSSV resistance in *P. monodon*.

**Results:**

A multiplex PCR system was developed to amplify the entire IHHNV genome, ensuring the accurate diagnosis. Field samples containing IHHNV DNA templates as low as 20 pg or equivalent 150 viral copies can be detected by this method. By challenging the two groups of diagnosed shrimp with WSSV, we found that shrimp with IHHNV infection and those with viral inserts responded to WSSV differently. Considering cumulative mortality, average time to death of shrimp in IHHNV-infected group (day 14) was significantly delayed relative to that (day 10) of IHHNV-inserted group. Real-time PCR analysis of WSSV copy number indicated the lower amount of WSSV in the IHHNV-infected group than the virus-inserted group. The ratio of IHHNV: WSSV copy number in all determined IHHNV-infected samples ranged from approximately 4 to 300-fold.

**Conclusion:**

The multiplex PCR assay developed herein proved optimal for convenient differentiation of shrimp specimens with real IHHNV infection and those with insert types. Diagnosed shrimp were also found to exhibit different WSSV tolerance. After exposed to WSSV, the naturally pre-infected IHHNV *P. monodon* were less susceptible to WSSV and, consequently, survived longer than the IHHNV-inserted shrimp.

## Background

The global shrimp aquaculture, with export values of billion dollars per year in the past decade, is a key economic sector of several countries in Asia and South America [1]. Despite overall shrimp production from aquaculture continuing to rise, shrimp susceptibility to viral pathogens is a constant threat to the shrimp production. Development of effective means that would reduce risks posed by viruses will be beneficial to the shrimp culture industry.

A shrimp parvovirus (densovirus) was first identified from high mortality disease outbreaks in cultured *Penaeus stylirostris* in the Americas [2], and it has been classified as *Penaeus stylirostris* densovirus (PstDNV) in the family *Parvoviridae*[3]. PstDNV is also known as infectious hypodermal and haematopoietic necrosis virus (IHHNV), and it will be referred to as IHHNV in the present work. Its genome contains a linear single-stranded DNA with an estimated size of 4.1 kb [4,5]. To date, 3 types of IHHNV have been reported based on their original found location and DNA sequences [6,7]. Target organs for IHHNV include gills, cuticular epithelium (or hypodermis), all connective tissues, haematopoietic tissues, lymphoid organ, antennal gland, ventral nerve cord - its branches and its ganglia [8].

More recently, non-infectious inserts of IHHNV in the shrimp genome have been found in both captured and farmed *P. monodon* from East Africa, Australia, and Thailand [9,10]. Recently, the study by Saksmerprome *et al.* (2011) indicated that random insertion of IHHNV sequences may yield false-positive results by using the currently recommended detection methods. Thus, it is necessary to improve the detection method for diagnosis of a real IHHNV infection. Besides the diagnostic implications, it is interesting to investigate if viral insertion in shrimp genome is involved in natural, transmissible immunity in crustaceans as previously proposed [11]. In addition, previous reports demonstrated that *P. vannamei* and *P. stylirostris* with pre-infection of IHHNV were more resistant to white spot syndrome virus (WSSV) as compared to the IHHNV-free shrimp [12,13], although the mechanism remains unknown. WSSV is one of the most severe viruses that could lead to mass mortalities in ponds and heavy production losses, therefore effective control strategies against the virus would be highly desirable. To elucidate the effect of persistent IHHNV infection and non-infectious viral inserts on WSSV resistance in *P. monodon*, a multiplex-PCR method developed herein was applied to conveniently distinguish between IHHNV-infected and IHHNV-inserted types. Then, the diagnosed shrimp with real IHHNV infection and viral inserts were examined for WSSV resistance by considering cumulative mortality, time-to-death, and WSSV copy number. Application of the multiplex PCR for selection of shrimp with viral tolerance could be useful for future development of a program of specific resistant (SPR) shrimp.

## Methods

### Multiplex PCR for differentiation of shrimp specimens with real IHHNV infection and those with insert types

A multiplex PCR-based method was developed to amplify the complete IHHNV genome in one step reaction via a two-tube method. The PCR assay was separated into 2 reaction mixtures. First reaction contained 3 primer pairs: F158-R723 (#1), F1451-R2355 (#2) and F3031-R3782 (#3), and the second reaction with 3 primer pairs: F702-R1578 (#4) and F2002-R3100 (#5), and Actin F/R (as an internal control). Primer sequences used in the assay were shown in Table 1. DNA templates were extracted from shrimp specimen with IHHNV-infected and IHHNV-inserted types, as previously determined [14]. PCR was conducted in a 25 μL reaction volume containing 2.5 μL PCR buffer (10X), 1 μL MgCl_2_ (50 mM), 0.5 μL dNTP (10 mM), 0.12 μL (1U) *Taq* DNA polymerase, 1 μL DNA template, and deionized water. The concentration used for all primer stocks was adjusted to 10 mM. For the first reaction, the amount used of each forward and reverse primer for primer set #1 was 0.1 μL, and for primer sets #2-3 was 0.3 μL. For the second reaction, 0.5 μL of each forward and reverse primers for primer sets #4-5 was used, while 0.25 μL actin-derived primers was added as an internal control amplification. With the ratio of 1:1, positive control mixture was composed of two plasmids, pCR-XL-TOPO (Invitrogen) with 3.6-kb IHHNV fragment and pDrive (QIAGEN) with IHHNV positioning 3031–3782 (GenBank AF273215). To determine the sensitivity of multiplex PCR, the PCR was repeated with amplification from 200 ng down to 2 pg of IHHNV-infected shrimp DNA extracted by phenol-chloroform method [15]. For analysis, 10 μL of the reaction were loaded onto each well and electrophoresed through a 1.5% w/v agarose gel in TAE buffer containing 0.5 g/L ethidium bromide. The detection sensitivity was also determined as a number of viral copies. Serial 10-fold dilutions of plasmid mixture were used as DNA template for each PCR reaction. The concentration of each plasmid DNA containing cloned PCR fragments covering entire IHHNV genome was determined by A_260/280_ absorbance values and used to calculate viral copy numbers with the following formula:

$$\text{Number}\;\text{of}\;\text{viral}\;\text{copy}=\frac{\text{Amount}\;\text{of}\;\text{DNA}\;\text{sample}\;\left(g\right)}{M.W.\;\text{of}\;\text{plasmids}\;\times \;1.66\;\times \;10^{-24}}$$

**Table 1:** Primers used in the multiplex PCR for the differentiation of IHHNV-infected and IHHNV-inserted types

**Primers (#)**	**Primer name**	**Sequences (5’ to 3’)**	**Product size (bp)**
1	F158	ATG GAA GAT ACG AAC AAC CA	566
	R723	GGA CCT GGG GTG AGA AGG CT	
2	F1451	GTT ACC TTT GCT GCC AGA GC	905
	R2355	GGA GGT ACC CAG TAG TCT ATA TC	
3	F3031	CTA AGG AAA CCG ACG TAA CA	752
	R3782	AAG TGA CGG CGG ACA ATA TC	
4	F702	CAA GCC TTC TCA CCC CAG G	877
	R1578	ATG GCG TGG CCA AGA C	
5	F2002	AGC TTG GAT AAT CAT CGT AGC AG	1099
	R3100	GCT GTT GAT TGT ACG GTC ACA AG	

### Shrimp and experimental WSSV infection

*P. monodon* shrimp (approximately 0.7 g) were cultured at Shrimp Genetic Improvement Center, Surat Thani province, Thailand. Since the Ethical Principles and Guidelines for the Use of Animals of the National Research Council of Thailand (1999) apply to vertebrates only and there is no official standard for invertebrates, we adapted its principles to shrimp. We also followed the guidelines of the Australian, New South Wales state government for the humane harvesting of fish and crustaceans

http://www.dpi.nsw.gov.au/agriculture/livestock/animal-welfare/general/fish/shellfish with respect to details regarding the transport of the shrimp and their laboratory maintenance. With respect to processing the shrimp for histological analysis or for killing at the end of an experiment, the salt water/ice slurry method was used as recommended in the Australian guidelines. Experimental shrimp were fed twice daily with a commercial feed at 3% (w/w), for 1 week prior the experiment. To evaluate the effects of pre-infection of IHHNV on subsequent WSSV infection, shrimp were divided into two experimental groups, pre-infected IHHNV (n=106) and IHHNV-free ones (n=108). Shrimp were exposed to WSSV by co-culturing with a WSSV-infected shrimp. Pleopods of each shrimp was dissected at the moribund stage at each time interval and then stored in 70% ethanol before delivered to Centex shrimp, Bangkok, for analyses. Mortalities were recorded twice a day and the “time to death post-challenge” was determined for each bioassay.

### Determination of IHHNV and WSSV copy number in challenged shrimp

All collected samples were subjected to genomic DNA extraction using the phenol-chloroform procedure as described previously [15]. DNA concentration in the extracts was quantified by spectrophotometry with A_260/280_ before adjustment to the concentration of 50 ng/μL in all assay. Real-time PCR was then performed in order to determine the copy number of both viruses in collected samples. The copy number of both IHHNV and WSSV were determined by using an external standard curve and plasmid DNA containing WSSV and IHHNV fragments as a standard (10-fold serial dilution, 10^7^-10^1^ copies). The copy number of analyses were calculated according to previous experiment [16]. Real-time PCR was operated on StepOnePlus cycler (Applied Biosystems, Life technology) using 2X TaqMan® Universal PCR Master Mix. Twenty times probe and primers mix used in the experiment were shown in Table 1. All probes and primers were designed according to reference GenBank accession numbers of AF273215 (IHHNV), AF440570.1 (WSSV) and DQ021452 (Shrimp elongation factor). The deionized water was used to adjust the final mixture volume to 20 μL. Amplification profile comprised of one initial step of 50°C 5 min then 95°C 15 min then followed by 40 cycles of 95°C for 15 s and 60°C for 1 min as required by Applied Biosystems. All data generated from real-time PCR was analyzed by StepOnePlus software 2.0 according to standard operation.

### Data analysis

PCR efficiency (E) for WSSV and IHHNV detection in this study was calculated by the equation of [E = 10^(−1/slope)^] [17] using cycle threshold (C_t_) [18] values fitted to a standard curve (WSSV; E=1.95, IHHNV; E=1.99). Statistical analysis of the viral copy number was carried out by one-way analysis of variance (ANOVA) with 95% confidence interval for mean using SPSS 16.0 software package.

## Results and discussion

### Two-tube multiplex PCR for differentiation of shrimp with IHHNV infection and those with IHHNV-inserted types

Here, we developed a multiplex PCR-based method that amplifies the complete IHHNV genome in one step via a two-tube method. In the case of samples with real infection, three and two bands were obtained from the first and second reactions, respectively (Additional file 1: Figure S1A-B). These results were expected, because the specimens were previously confirmed as being infected by IHHNV and should have the entire genome detected by primers in the multiplex method. Samples with non-infectious (inserts) gave incomplete results, while shrimp actin amplicon appeared in all lanes (Additional file 1: Figure S2A-B). Absence of any of the five DNA fragments was interpreted as a negative result with putative viral inserts. By agarose gel electrophoresis, the lowest amount of DNA template that gives visible PCR products for the two reaction was approximately 20 pg. The detection sensitivity of the multiplex PCR system was estimated to be 150 viral copy equivalents (Additional file 1: Figure S3A-B).

It is possible that shrimp carrying non-infectious inserts are infected by the virus. If it is the case, the multiplex PCR may not be able to separate such specimens from those with IHHNV infection only. Nevertheless, the developed method remains useful for selection of specific pathogen-free shrimp. It also serves the main purpose of the study for investigating the effect of persistent IHHNV infection of WSSV resistance, regardless of the presence of non-infectious inserts within individuals.

### Different responses to WSSV challenge of IHHNV pre-infected and IHHNV-inserted *P. monodon*

**Figure 1:**
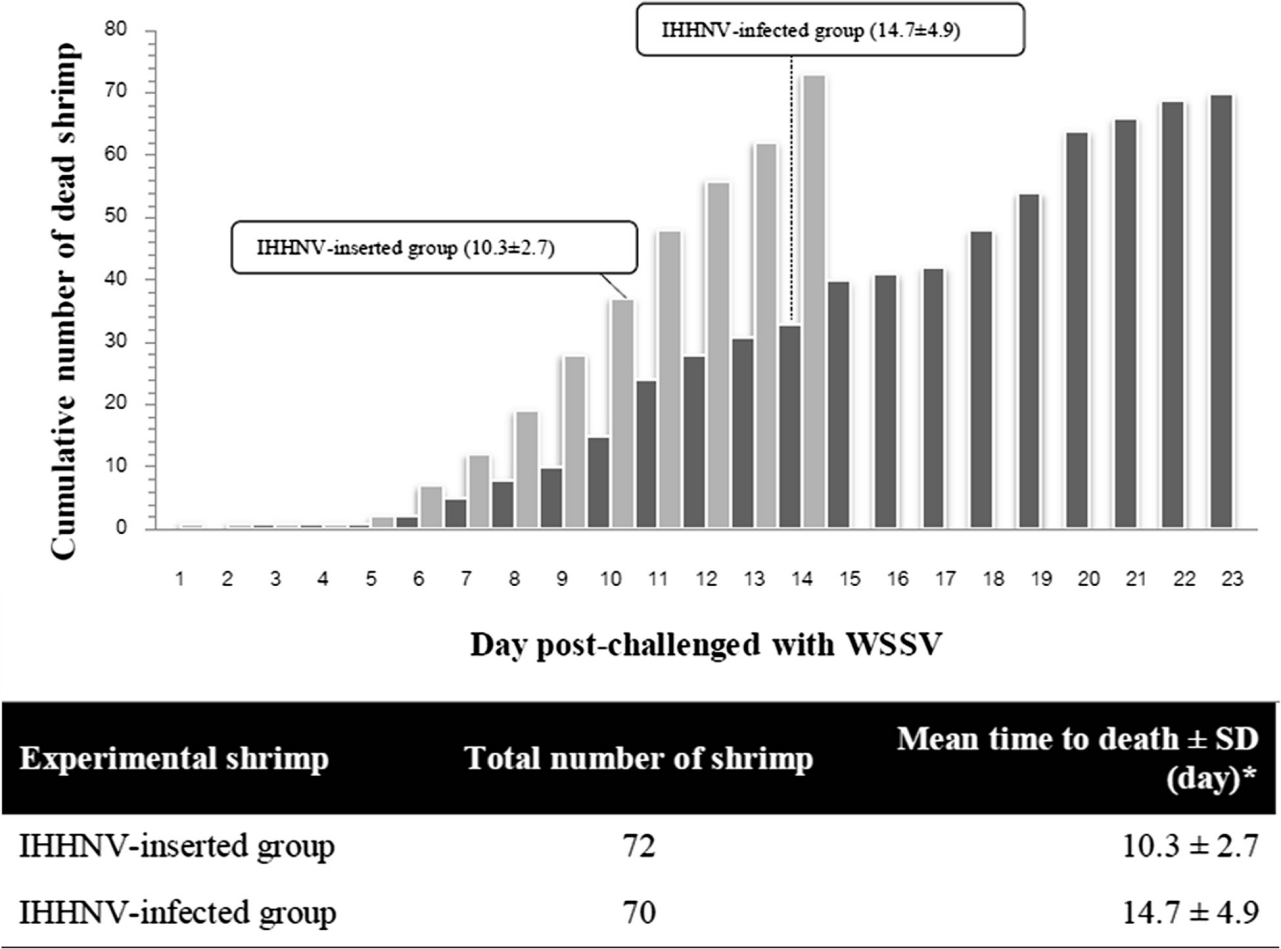
**A graph of cumulative mortality of experimental shrimp after challenging with WSSV (top) and a table of calculated time to death data between IHHNV-inserted and IHHNV-infected group (bottom).** Data was analyzed by ANOVA analysis using SPSS16.0 software. Asterisk (*) indicated the significant difference between mean of time to death from IHHNV-inserted and IHHNV-infected group with *P*<0.05. Grey and black columns indicated IHHNV-inserted and IHHNV-infected groups, respectively. (Uploaded separately).

Experimental shrimp were divided into 2 groups based on the results of two-tube multiplex PCR detection method as described above. The first group was the IHHNV-infected group and the second was the IHHNV–inserted group (Table 2). Figure 1 shows a cumulative mortality of experimental shrimp after WSSV challenged. The average mean time to death ± standard deviation (SD) from these two groups are 10.3 ± 2.7 d; IHHNV-inserted group and 14.7 ± 4.9 d; IHHNV-infected group. Statistical analysis using SPSS 16.0 software (ANOVA) indicated that these two calculated values are significantly different (*P*<0.05).

**Table 2:** ***P. monodon *shrimp used in the study and the characterization of samples by Multiplex PCR**

**Sample no.**	**Multiplex MI**	**Multiplex MII**	**IHHNV-inserted type**	**IHHNV-infected type**
6	+	-	√	
7	+	-	√	
8	+	-	√	
9	+	-	√	
10	+	-	√	
12	+	-	√	
14	+	-	√	
15	+	-	√	
18	+++	++		√
19	+++	++		√
20	+++	++		√
21	+++	++		√
22	+++	++		√
23	+++	++		√
24	+++	++		√
25	+++	++		√
27	+++	++		√
28	+++	++		√
29	+++	++		√

Multiplex MI should contained 3 PCR products (+ + +) and Multiplex MII should contained 2 PCR products (+ +). Mark (√) indicated the group for each sample after characterization by Multiplex PCR.

A real-time PCR analysis using TaqMan® MGB Probe was performed to determine the number of viral copies of WSSV in shrimp from the two groups. Eight samples from the inserted group and eleven samples from the infected group were chosen, and then they were examined for DNA quality by amplification of shrimp housekeeping gene (shrimp elongation factor) using specific TaqMan® MGB Probe (Table 3). Determination of IHHNV and WSSV copy number was performed as described in materials and methods. The estimated mean ± SD of WSSV copies were 1.74 × 10^7^ ± 1.78 × 10^7^ copies for IHHNV-inserted group, and 2.74 × 10^6^ ± 2.41 × 10^6^ copies for IHHNV-infected group. Statistical analysis using ANOVA suggested that mean of WSSV copies from IHHNV-infected samples was significantly lower than those estimated from IHHNV-inserted samples (*P*<0.05).

**Table 3:** Primers and probes used in real-time PCR analysis

**Primer name**	**Sequence (5’to 3’)**	**Detection**
WS440570.1-F	CAA CTA GCA AGC ACT GGT ATT TTC AAT AA	
WS440570.1-R	CGG CCA ATA AAT CTG CAG TCA TT	WSSV
WS440570.1-Probe	CAA CAT GCC AGT TTT C	
IH273215-F	ACT ACG ACA ATA TGC AGT GGA TAA AAT ACA	
IH273215-R	GTC TGC TAC GAT GAT TAT CCA AGC T	IHHNV
IH273215-Probe	CCG TGT ACC AGA AAT C	
EF021452-F	CGT GAA GAA CGT GTC TGT AAA GGA T	
EF021452-R	GGC TGG GTC GTT CTT CGA	Shrimp elongation factor
EF021452-Probe	AAG CGA CGA ATC CAC	

As mentioned above, some infected specimen may carry non-infectious inserts. If the viral inserts present in the shrimp were primed by the PCR primers used for quantification, the number of IHHNV copies per shrimp in the infected group could be overestimated. Unknown overestimations due to random inserts might explain a wide range of IHHNV copies per shrimp (from 8.02 × 10^5^ to 4.28 × 10^8^ copies), and the resulting IHHNV-to-WSSV ratios varied from 4- to 300-fold in IHHNV-infected samples (Table 4). Nonetheless, this does not alter the fact that WSSV copies in IHHNV-infected samples were much lower than those in the inserted ones. Whether the persistence of IHHNV infection outcompete WSSV entry/replication in the infected shrimp should be further investigated by additional techniques, such as *in situ* hybridization.

**Table 4:** Different ratios of determined IHHNV to WSSV copy number in the IHHNV- infected samples

**Group of sample**	**Sample no.**	WSSV titres*	**IHHNV titres**	**Ratio of IHHNV: WSSV**
IHHNV-inserted group	6	5.76E+07	NA	-
	7	7.15E+05	NA	-
	8	1.08E+07	NA	-
	9	2.09E+07	NA	-
	10	1.64E+07	NA	-
	12	1.07E+06	NA	-
	14	1.59E+07	NA	-
	15	1.62E+07	NA	-
	Mean±SD	1.74E+07±1.78E+07	NA	-
IHHNV-infected group	18	2.32E+05	1.85E+07	79.8
	19	5.54E+06	4.77E+07	8.6
	20	4.36E+05	8.75E+07	200.6
	21	3.82E+06	5.11E+07	13.4
	22	7.54E+06	1.62E+08	21.5
	23	4.38E+06	1.94E+07	4.4
	24	8.77E+05	7.07E+07	80.6
	25	2.24E+06	1.06E+08	47.2
	27	2.10E+06	7.18E+07	34.2
	28	2.96E+06	4.28E+08	144.6
	29	2.66E+03	8.02E+05	301.4
	Mean±SD	2.74E+06±2.41E+06	9.67E+07±1.19E+08	85.12±94.49

Asterisk (*) indicated that mean from these two groups were significant difference with *P*<0.05, *NA*= not applicable.

Our findings suggested that shrimp with pre-infection of IHHNV exhibited a longer survival time after exposure to WSSV as compared to survival time of shrimp with IHHNV inserts. This is the first report on pre-infection with IHHNV delaying mortality induced by WSSV in *P. monodon* shrimp. Real-time PCR results revealed that IHHNV existed at a higher copy number relative to WSSV copy number in samples with infectious IHHNV, ranging from 4 to 300-fold. Our results were similar to those reported from *P. stylirostris*[13] and *P. vannamei*[12]. Persistence of IHHNV may block the entry of another virus by mechanism of down-regulation of viral receptor(s) or by competition for receptor binding [12]. Moreover, target cells of IHHNV are hypodermal and mesodermal cells, whereas those of WSSV are ectodermal and mesodermal cells [19,20]. If persistence of IHHNV is mainly in mesodermal cells, and it could out-complete WSSV replication in the infected cells. Therefore, the effects of WSSV infection in diseased shrimp, in turn, could be reduced. Future work should include investigating infected cells, specifically mesodermal cells, for possible viral interference between IHHNV and WSSV.

## Conclusions

Persistent IHHNV infection and non-infectious viral inserts in *P. monodon* can be commonly found, and it is interesting to elucidate any implication of their existence in nature. IHHNV pre-infected *P. vannamei* and *P. stylirostris* were reported to survive longer than non-infected ones after WSSV challenge [12,13], however the detailed mechanism is not well-understood. In this study, a multiplex PCR method was developed to distinguish *P. monodon* with persistent IHHNV infection from those with viral inserts. Field samples containing IHHNV DNA templates, as low as 20 pg or equivalent of 150 viral copies can be determined by this method. While the multiplex PCR system could be used independently, use of the multiplex PCR as an additional detection procedure would ensure a more accurate diagnosis of a real infectious IHHNV by ruling out the possibility of viral genomic inserts in shrimp specimens. Finally, the method allowed us to conveniently prepare samples for studying the effect of pre-infection of IHHNV, in comparison to IHHNV inserts, on WSSV resistance in *P. monodon*. Our preliminary work suggests that persistent IHHNV infection could delay WSSV-induced mortality in *P. monodon*.

## Competing interest

The authors declare that they have no competing interest.

## Authors’ contributions

This work has been done at Centex shrimp, Faculty of Science, Mahidol University and Shrimp Genetics Improvement Center, Surat Thani, Thailand. Experimental shrimp were prepared and challenged by the assistance of BW and PP’s group. The method for differentiate between IHHNV inserts and real IHHNV infected samples was discovered by VS with the kind help of SJ and YJ. Viral copy number, real-time PCR work including data analysis and manuscript preparation was done by SM and VS under the supervision of CB, TW and BW. All authors read and approved the final manuscript.

## Supplementary Material

Additional file 1: Figure S1 Agarose gels showing DNA pattern of positive samples from the first multiplex reaction with 3 IHHNV primers sets (A) and the second reaction with 2 IHHNV primer sets with an actin-derived primer pair (B). Lanes 1 and 8, DNA ladder; 2, positive control (plasmids containing complete IHHNV genome); 3-6, individual shrimp samples; 7, negative control. **Figure S2.** Agarose gels showing DNA pattern of samples with putative viral inserts from the first multiplex reaction (A) and the second reaction (B). Lanes 1 and 7, DNA ladder; 2, positive control (plasmids containing entire IHHNV genome); 3-5, individual shrimp samples; 6, negative control. **Figure S3.** Sensitivity analysis of the first multiplex reaction in (A), and the second reaction in (B). Lanes 1 and 10, DNA ladder; 2, positive control; 3-8, the amount of DNA template varying from 200 ng, 20 ng, 2 ng, 200 pg, 20 pg, 2 pg, respectively; 9, negative. Click here for file
