# Supplementary material for: Persistence of Penaeus stylirostris densovirus delays mortality caused by white spot syndrome virus infection in black tiger shrimp (Penaeus monodon)
Source: BMC Vet Res. 2013 Feb 15;9:33. doi: 10.1186/1746-6148-9-33 (PMC3598937; doi:10.1186/1746-6148-9-33)

Additional figures

Additional figure 1: Agarose gels showing DNA pattern of positive samples from the first multiplex reaction with 3 IHHNV primers sets (A) and the second reaction with 2 IHHNV primer sets with an actin-derived primer pair (B). Lanes 1 and 8, DNA ladder; 2, positive control (plasmids containing complete IHHNV genome); 3-6, individual shrimp samples; 7, negative control

Additional figure 2: Agarose gels showing DNA pattern of samples with putative viral inserts from the first multiplex reaction (A) and the second reaction (B). Lanes 1 and 7, DNA ladder; 2, positive control (plasmids containing entire IHHNV genome); 3-5, individual shrimp samples; 6, negative control

Additional figure 3: Sensitivity analysis of the first multiplex reaction in (A), and the second reaction in (B). Lanes 1 and 10, DNA ladder; 2 , positive control; 3-8, the amount of DNA template varying from 200 ng, 20 ng, 2 ng, 200 pg, 20 pg, 2 pg, respectively; 9, negative.

Additional figures (cont.)

Additional figure 1

A)

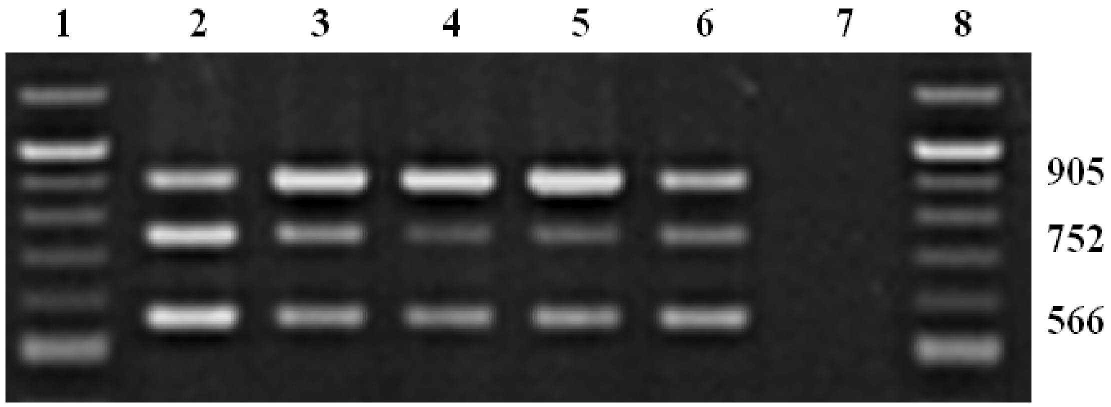

B)

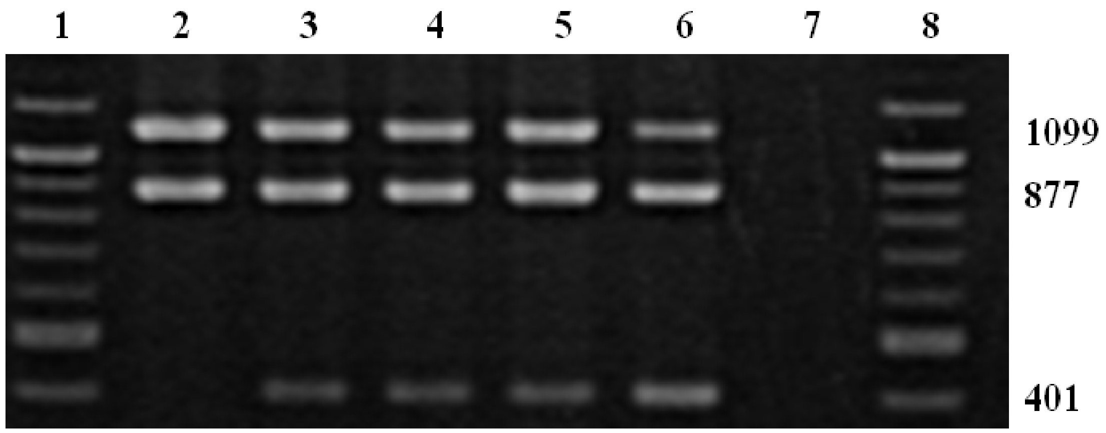

Additional figure 2

A)

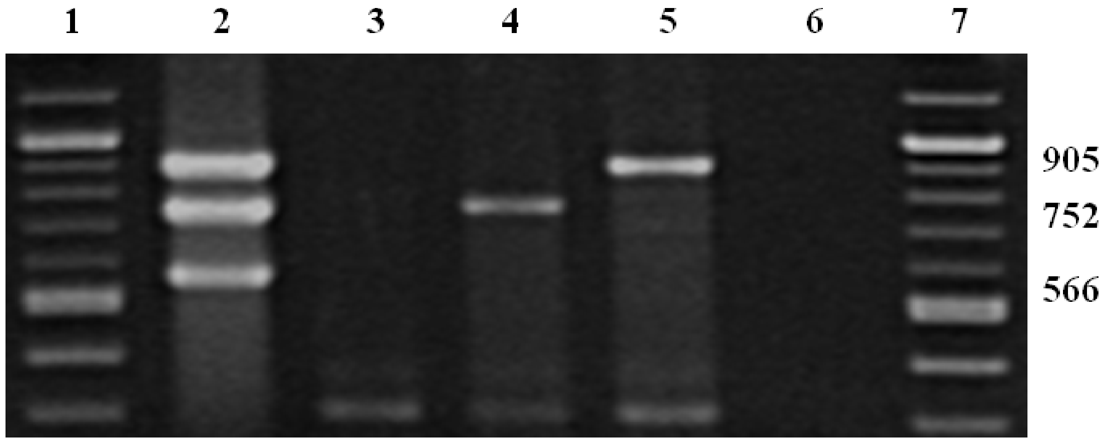

B)

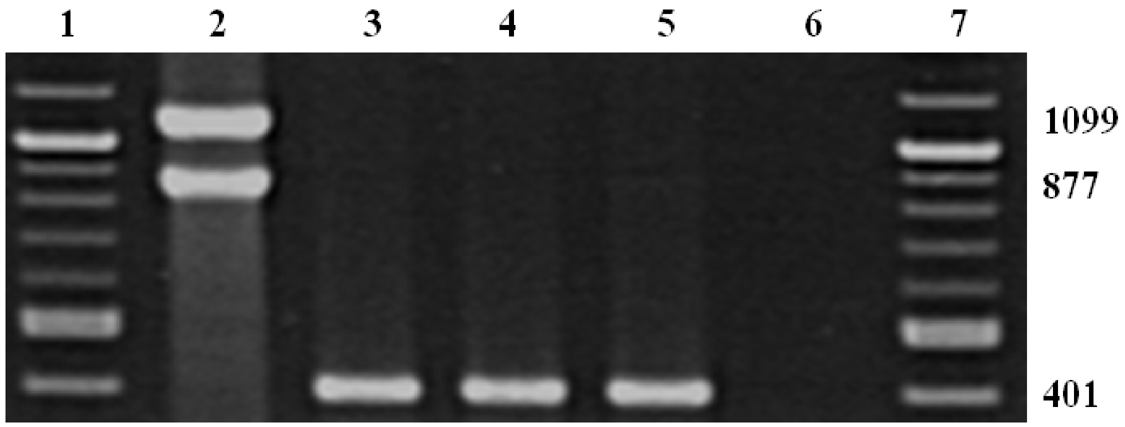

Additional figure 3

A)

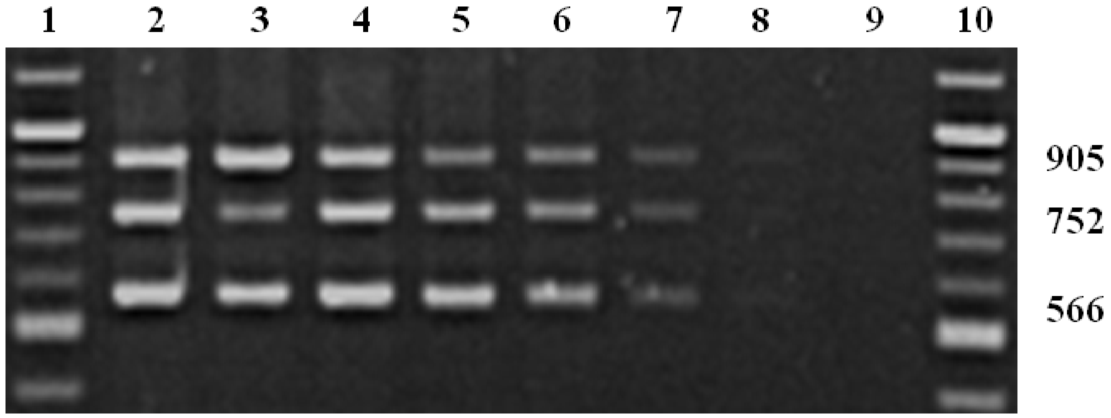

B)

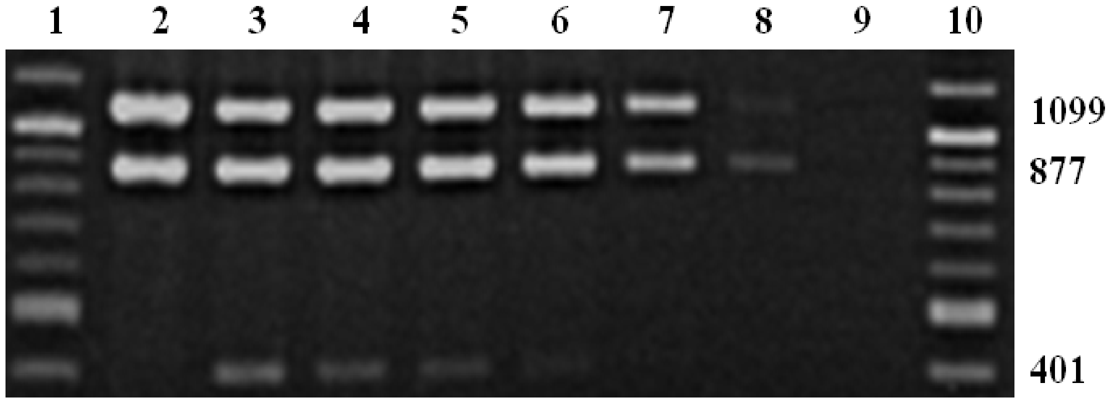

Supplement: Additional file 1: Figure S1 — Agarose gels showing DNA pattern of positive samples from the first multiplex reaction with 3 IHHNV primers sets (A) and the second reaction with 2 IHHNV primer sets with an actin-derived primer pair (B). Lanes 1 and 8, DNA ladder; 2, positive control (plasmids containing complete IHHNV genome); 3-6, individual shrimp samples; 7, negative control. Figure S2. Agarose gels showing DNA pattern of samples with putative viral inserts from the first multiplex reaction (A) and the second reaction (B). Lanes 1 and 7, DNA ladder; 2, positive control (plasmids containing entire IHHNV genome); 3-5, individual shrimp samples; 6, negative control. Figure S3. Sensitivity analysis of the first multiplex reaction in (A), and the second reaction in (B). Lanes 1 and 10, DNA ladder; 2, positive control; 3-8, the amount of DNA template varying from 200 ng, 20 ng, 2 ng, 200 pg, 20 pg, 2 pg, respectively; 9, negative. [file 1746-6148-9-33-S1.pdf]
